# Magnetic, Acceleration Fields and Gyroscope Quaternion (MAGYQ)-Based Attitude Estimation with Smartphone Sensors for Indoor Pedestrian Navigation

**DOI:** 10.3390/s141222864

**Published:** 2014-12-02

**Authors:** Valérie Renaudin, Christophe Combettes

**Affiliations:** IFSTTAR, GEOLOC Laboratory, Route de Bouaye CS4, Bouguenais 44344, France; E-Mail: christophe.combettes@ifsttar.fr

**Keywords:** indoor navigation, MEMS, quaternion, attitude estimation, Kalman filter, magnetic angular rate update, acceleration gradient update, magnetometer

## Abstract

The dependence of proposed pedestrian navigation solutions on a dedicated infrastructure is a limiting factor to the deployment of location based services. Consequently self-contained Pedestrian Dead-Reckoning (PDR) approaches are gaining interest for autonomous navigation. Even if the quality of low cost inertial sensors and magnetometers has strongly improved, processing noisy sensor signals combined with high hand dynamics remains a challenge. Estimating accurate attitude angles for achieving long term positioning accuracy is targeted in this work. A new Magnetic, Acceleration fields and GYroscope Quaternion (MAGYQ)-based attitude angles estimation filter is proposed and demonstrated with handheld sensors. It benefits from a gyroscope signal modelling in the quaternion set and two new opportunistic updates: magnetic angular rate update (MARU) and acceleration gradient update (AGU). MAGYQ filter performances are assessed indoors, outdoors, with dynamic and static motion conditions. The heading error, using only the inertial solution, is found to be less than 10° after 1.5 km walking. The performance is also evaluated in the positioning domain with trajectories computed following a PDR strategy.

## 1. Introduction

The advent of new wearable devices has widened the application field of pedestrian navigation systems and methods. Among these devices, handheld units remain of prime interest for Location Based Services (LBS) [1]. Existing technologies are still lacking in terms of positioning and navigation performance. Either they depend on a dedicated infrastructure, which is not continuously available during pedestrian journeys introducing coverage gaps, or the accuracy of the position estimate is not sufficient for locating the user on the correct street location (sidewalk, stairway, *etc.*). The future of pedestrian navigation solutions certainly relies on combining all existing technologies depending on the user's context. Finding the appropriate criteria for shaping the hybridization filter remains a great challenge.

**Figure 1.**
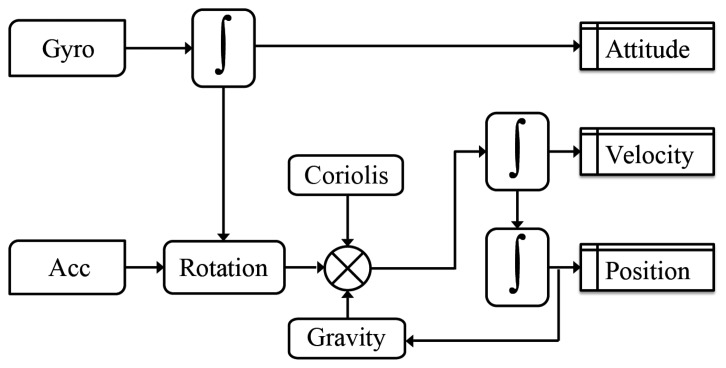
Simplified flowchart of strapdown mechanization equations.

**Figure 2.**
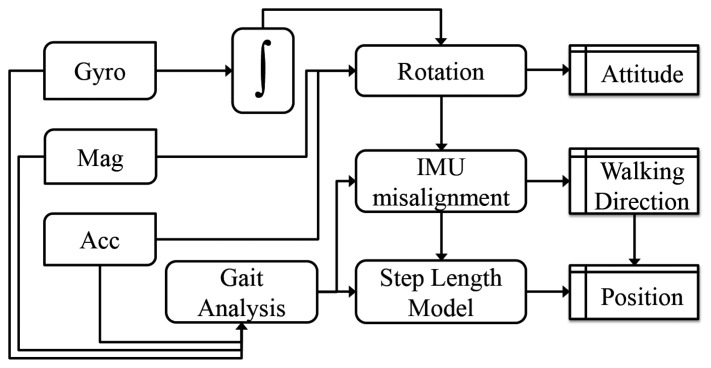
Pedestrian Dead Reckoning (PDR) mechanization equations with handheld tri-axis inertial sensors and tri-axis magnetometers.

One option consists in developing self-contained sensor navigation solutions. Still today, the quality of inertial sensors embedded in smartphone devices is too low for solving all free-inertial pedestrian navigation issues. This statement is true irrespective of the adopted processing strategy [2]: inertial strapdown navigation (Figure 1) or pedestrian dead-reckoning (Figure 2). However the latest performance improvements of micro-electro-mechanical sensors (MEMS) combined with novel algorithms for mitigating the sensor errors using opportunistic signals are pushing the boundaries of existing inertial pedestrian navigation solutions.

Whether using a strapdown inertial navigation method or a pedestrian dead-reckoning one, the attitude angles, including the heading, are estimated by integrating the angular rates sensed by a gyroscope. With this approach, the gyroscope errors are propagated at a time cubic rate. With low cost handheld units, the options for mitigating the sensors drift and noise are limited. Indeed, as compared to what would have been applied with foot mounted systems during the stance phases of the walking gait, no zero angular rate or zero velocity update can be applied. With respect to magnetic fields, no geomagnetic heading update can be easily and frequently applied in urban and indoor spaces because the earth magnetic field is strongly perturbed by surrounding artificial fields [3].

Another source of error comes from the parameterization of the rotation between the mobile unit and the navigation frame. It is known that Euler parameterization in attitude angles estimation filter introduces gimbal lock problems. Because the hand performs fast motions in the entire 3D space without privileging any direction, frequent occurrence of this issue is observed. With free inertial pedestrian navigation based on handheld device comes another problem: the time varying relative orientation between the reference frame of the handheld device and the one related to the pedestrian's body. Existing methods [4,5] propose to solve this issue using the accelerations in the navigation frame, which reinforces the critical role of accurate attitude angles estimation in the overall PDR accuracy budget. All these factors highlight the critical role of heading estimation in the overall performance of inertial pedestrian navigation solutions with handheld smartphone. It definitively motivates the research work presented in this article.

In this paper, a novel Magnetic, Acceleration fields and GYroscope Quaternion (MAGYQ)-based attitude and heading estimation filter is proposed and demonstrated with handheld sensors. First, the filter takes advantages of the four-dimensional quaternion algebra for reducing the errors introduced by the mathematical representation of rotations and uses a quaternion based state vector. Second, it exploits specific states of the measured magnetic field and acceleration vector combined with some hand motions of opportunity for observing the attitude angles and mitigating the sensor errors.

Section 2 starts with a state-of-the-art on existing attitude estimation solutions and continues with the innovations that are proposed in this article. Theoretical aspects of the novel quaternion error-based attitude heading estimation method including a new angular rate signal model are detailed in Section 3. The algorithms involved in the attitude angle estimation filter MAGYQ are detailed in Sections 4–6. MAGYQ headings are combined with step lengths that are computed using a model recalled in Section 7. Section 8 is dedicated to the experimental assessment. A performance analysis of the attitude angles estimation is first conducted and followed by an evaluation in the positioning domain.

## 2. State of the Art

### 2.1. Existing Attitude Estimation Approaches

Existing attitude and heading estimation filters are principally based on the use of Direct Cosine Matrix (DCM) parameterized with Euler angles. It describes the transformation between coordinate frames that are linked to the handheld unit: the body frame and a local navigation frame. Tri-axis gyroscope, tri-axis accelerometer and tri-axis magnetometer are the most common sensors used for estimating this rotation.

#### 2.1.1. Euler Angles with Direct Cosine Matrix

Classically small rotation errors are tracked using Euler parameterization for estimating the attitude rotation matrix [6]. The inherited algorithms use Euler angles to describe the three dimensional rotation and the three successive rotations (yaw ψ, pitch θ, and roll φ). The DCM ${\text{C}}_{b}^{n}$ is parameterized using Euler angles and defines the rotation between the body frame and the navigation (or local) frame. Unfortunately, DCM parameterized with Euler angles presents singularities, which are known as the “Gimbal lock” problem. If the pitch angle equals π/2, several pairs of yaw and roll angles can achieve the same rotation. Furthermore this parameterization is highly non-linear since the DCM is composed of sums of products of sine and cosine functions. Therefore proposing an attitude angles estimator that remains in the quaternion set is of interest to overcome these limitations.

#### 2.1.2. Coupling of Magnetic Field and Inertial Measurements

Many filters have been proposed for improving the attitude estimation using magnetic field measurement. They are based on hybridization techniques that use either *a priori* knowledge of the local magnetic field or at least its properties. Irrespective of the chosen approach, they all require a magnetometer calibration for removing the magnetic field induced by the platform [7] in order to measure only the surrounding magnetic field. Four different main magnetic field based methods, which have been proposed in the literature, are now recalled:

- Searching for the geomagnetic earth field
- Fingerprinting with magnetic anomalies
- Sensing the velocity with a magnetometer array
- Magnetic angular rate update with DCM

Let **B***^n^* be the known local magnetic field in the navigation frame. This field is related to the magnetic field **B***^b^* expressed in the body frame by the DCM with:

(1) $$B^{n}=C_{b}^{n}B^{b}$$

##### Searching for the Geomagnetic Earth Field

The first method tries to remove all artificial sources of magnetic field that are perturbing the known Earth magnetic field [8]. This approach involves geomagnetic field detectors that often fail to correctly discriminate magnetic perturbations from the geomagnetic field. This is especially the case in indoor spaces, where many artificial magnetic field sources modify the Earth field [3]. This method is often combined with pitch and roll angles estimation using accelerations that are measured during static phase of the inertial mobile unit (IMU). During these periods, the accelerometer measures the Earth gravity directly. Combining the measurement of these two fields, it becomes possible to rebuild the complete orientation following for example the QUEST [9] or MARG [10,11] algorithms.

##### Fingerprinting with Magnetic Anomalies

A more recent method, which is growing in popularity, consists in navigating using only measured magnetic fields. At first, the indoor (even outdoor) magnetic fields are mapped during a calibration phase [12,13]. Following Wi-Fi receiver signal strength based fingerprinting techniques, the user's location is extracted from the database of magnetic anomalies. This method benefits from the singularities in the local magnetic field anomalies, but it is non-ergonomic and laborious. Up to now, its limitations have hardly been investigated. One limitation among others is the influence of soft-iron effects in a crowded environment, which has not yet been assessed.

##### Sensing the Velocity with a Magnetometer Array

Another approach, frequently applied to robotics, uses array of magnetometers for measuring the magnetic field gradient and deducting the velocity vector [14]. No a priori knowledge about the local magnetic field is required for this approach where the magnetic field space gradient (Δ**B**^b^/Δ**x**) and the derivate of the position in the body frame (Δ**x**/Δ*t*) are related to the magnetic field temporal derivate (Δ**B**^b^/Δ*t*) by:

(2) $$\frac{\Delta B^{b}}{\Delta t}=\frac{\Delta x}{\Delta t}\frac{\Delta B^{b}}{\Delta x}$$

The biggest challenge of this method comes from the individual calibration of all magnetometers since the precision of the measured gradient directly impacts the estimated velocity. This estimation becomes really difficult with varying fields on the hosting platform. Finally, the distance between each magnetometer strongly impacts the good approximation of the space gradient, which might be a limiting factor for handheld sensors.

##### Magnetic Angular Rate Update with DCM

The last technique exploits Quasi-Static magnetic Field (QSF) in the navigation frame for mitigating gyroscope error. This approach works even if the sensed field differs from the earth geomagnetic field [15]. An important feature of this method is its immunity to local magnetic field disturbances, as long as they are globally constant in the local frame. If this hypothesis holds, the magnetic field temporal gradient gives a direct observation of the body frame angular rate (ω):

(3) $$\frac{dB^{b}}{dt}=-\omega \wedge B^{b}$$

∧ is the cross product of two vectors. The novel MAGYQ filter proposed in this paper is based on this approach. Let us note that unlike previous magnetometer array-based algorithms, it uses only one tri-axis magnetometer.

### 2.2. Innovation of the Proposed Method

The first innovation is that the Magnetic, Acceleration fields and GYroscope Quaternion (MAGYQ)-based attitude and heading estimation filter uses quaternions to parameterize the state vector and the angular rates measurements. Instead of using Euler angle errors or its quaternion form, MAGYQ filter estimates an additive quaternion error. A new gyroscope signal model in the quaternion set is also proposed and a gyroscope quaternion bias is introduced. The filtering strategy is based on an Extended Kalman Filter (EKF) whose aim is to estimate the attitude of the body frame of a handheld device with respect to a local frame. In Section 3, this novel approach of angular rate modelling in the quaternion set is detailed and justified for the Kalman Filter underlying hypothesis.

The second innovation comes from the update steps. Observation equations are proposed using the magnetic field angular rate (MARU) in the quaternion set and using the acceleration gradient update (AGU). The benefit of MARU is that it can frequently be applied for bounding the gyroscope errors even in indoor spaces where the Earth magnetic field is disturbed. The accelerometer gradient update reduces the error propagation and estimates the accelerometer bias components. In the literature, the Earth gravity field is used as a local reference field during static periods. It is here proposed to go beyond this approach. Similarly to MARU, the rotation of the IMU is observable using the accelerometer signal gradient if no lever arm between the triads of gyroscopes and the accelerometers exists. AGU takes advantage of opportune hand motions for mitigating the accelerometer errors but also observing the gyroscope bias.

## 3. Gyroscope Quaternion Modelling

The proposed attitude estimation algorithm is based on angular rates and accelerations sensed with a handheld low cost inertial mobile unit. Contrary to existing solutions, however, the gyroscope error modeling is not performed in the signal domain but in the quaternion set. This approach should improve the accuracy of the estimated angles thanks to reduced linearization steps and ambiguity issues. Prior to presenting the quaternion based error modeling, main properties of the quaternion set used for expressing the rotations are recalled.

### 3.1. Quaternion Algebra

This part recalls some quaternion properties and the different notations used in this paper. Quaternions are used as parameters for estimating the orientation of a rigid body [9,16].

#### 3.1.1. General Content

The set of quaternion real numbers, noted ℍ in tribute to the mathematician William Rowan Hamilton, is a fourth dimensional non-commutative algebra including specific composition laws [17]. An element **q** ∈ ℍ is defined with a real-quadruplet (*q*_1_,*q*_2_,*q*_3_,*q*_4_). Each quaternion **q** can be expressed as a unique pair (*q*_1_,**u**_q_) in which *q*_1_ is the scalar part and ${u_{\text{q}}}^{T}=\left(\begin{array}{ccc} q_{2} & q_{3} & q_{4} \end{array}\right)$ is the vector or imaginary part. In this paper, the quaternion multiplication is noted ⊗. In the scalar-vector form, the product of two quaternions **x** and **y**, noted **x** ⊗ **y**, is:

(4) $$\left(\begin{array}{c} x_{1}\\ u_{x} \end{array}\right)⊗\left(\begin{array}{c} y_{1}\\ u_{y} \end{array}\right)=\left(\begin{array}{c} x_{1}y_{1}-\langle u_{x},u_{y}\rangle \\ x_{1}u_{y}+y_{1}u_{x}+u_{x}\wedge u_{y} \end{array}\right)$$

with ^ the cross product and <,> the classic scalar product in ℝ^3^. A second form to represent the quaternion multiplication is to use the matrix product:

(5) $$\begin{array}{cc} \begin{array}{ll} \text{x}⊗\text{y} & =\text{M}\left(\text{x}\right)\text{y}\\ & =\left(\begin{array}{cccc} {\text{x}}_{1} & -{\text{x}}_{2} & -x_{3} & -x_{4}\\ x_{2} & x_{1} & -x_{4} & x_{3}\\ x_{3} & x_{4} & x_{1} & -x_{2}\\ x_{4} & -x_{3} & x_{2} & x_{1} \end{array}\right){\left(\begin{array}{c} {\text{y}}_{1}\\ {\text{y}}_{2}\\ y_{3}\\ y_{4} \end{array}\right)}_{\text{or}} \end{array} & \begin{array}{ll} x⊗y & =\text{C}\left(y\right)x\\ & =\left(\begin{array}{cccc} y_{1} & -y_{2} & -y_{3} & -y_{4}\\ y_{2} & y_{1} & y_{4} & -y_{3}\\ y_{3} & -y_{4} & y_{1} & y_{2}\\ y_{4} & y_{3} & -y_{2} & y_{1} \end{array}\right)\left(\begin{array}{c} x_{1}\\ x_{2}\\ x_{3}\\ x_{4} \end{array}\right) \end{array} \end{array}$$

Other properties such as conjugation and norm are defined in the quaternion set. For each quaternion **q**, there exists a unique conjugate noted **q̅** and a norm ‖**q**‖ defined by:

(6) $$\left\{ \begin{array}{l} \bar{q}=\left(\begin{array}{c} q_{1}\\ -u_{q} \end{array}\right)\\ \left\|q\right\|=\sqrt{{q_{1}}^{2}+{q_{2}}^{2}+{q_{3}}^{2}+{q_{4}}^{2}}=\sqrt{{q_{1}}^{2}+{\left\|u_{q}\right\|}^{2}} \end{array}\right.$$

For a vector **x** ∈ ℝ^3^, a unique quaternion form is defined and links the quaternion set to the three dimensional space by:

(7) $${\left(x\right)}_{q}=\left(\begin{array}{c} 0\\ x \end{array}\right)$$

#### 3.1.2. Quaternion and Rotation

A unit quaternion **q** can be rewritten with a pair (θ, **u**) with **u** a unit vector and θ∈]− π, π]:

(8) $$q=\left(\begin{array}{c} \cos\theta \\ \sin\theta u \end{array}\right)$$

The quaternion defined in Equation (8) describes a rotation [18] in the three dimensional space. **u** is the rotation axis and θ the rotation angle. By specifying the direction axes, there exists a unique unit quaternion noted $q_{a}^{b}$ that represents the rotation from frame *a* to frame *b*. The vector **x** expressed in frame *a*, can be computed in frame *b* with:

(9) $${\left(x^{b}\right)}_{q}=q_{a}^{b}⊗{\left(x^{a}\right)}_{q}⊗{\bar{q}}_{a}^{b}$$

Similarly to the rotational matrix ${\text{C}}_{a}^{b}$, expressed with Euler angles roll-pitch-yaw (φ,θ,ψ) sequences, quaternion offers another parameterization of a rotation. Quaternion differential equation can also be used for expressing the evolution of the rotation between the two frames *a* and *b*. The rotational quaternion derivative noted ${\dot{q}}_{a}^{b}$ is given by:

(10) $${\dot{q}}_{a}^{b}=\frac{1}{2}q_{a}^{b}⊗{\left(\omega_{ba}^{a}\right)}_{q}$$

where $\omega_{ba}^{a}$ is the angular rate of frame *b* with respect to frame *a*, expressed in the frame *a*.

### 3.2. Sensor Signal Modelling

Because the sensors are of MEMS quality, signal models are proposed for each sensor: tri-axis magnetometer, tri-axis accelerometer and tri-axis gyroscope. All sensor frames are assumed to be orthogonal, co-aligned with the same origin. Consequently, a unique sensor frame for all sensors is defined and labelled body frame with the symbol *b*. This part presents the error model chosen for each sensor: at first the magnetometer, then the accelerometer and finally the gyroscope.

#### 3.2.1. Magnetometer

The magnetometer measures the local magnetic field in the body frame. In order to remove all sensor perturbation sources: scale factor, non-orthogonally, bias and magnetic deviation due to the hosting platform, a calibration phase is first realized [19]. Only white noise remains:

(11) $$y_{m}^{b}=m^{b}+n_{m}$$

where **y***_m_* is the magnetometer signal, **m***^b^* is the local magnetic field expressed in the body frame. It includes the earth magnetic field and the local disturbances. The zero mean Gaussian white noise process is noted **n***_m_* and characterized by a standard deviation σ*_m_*.

#### 3.2.2. Accelerometer

**Figure 3.**
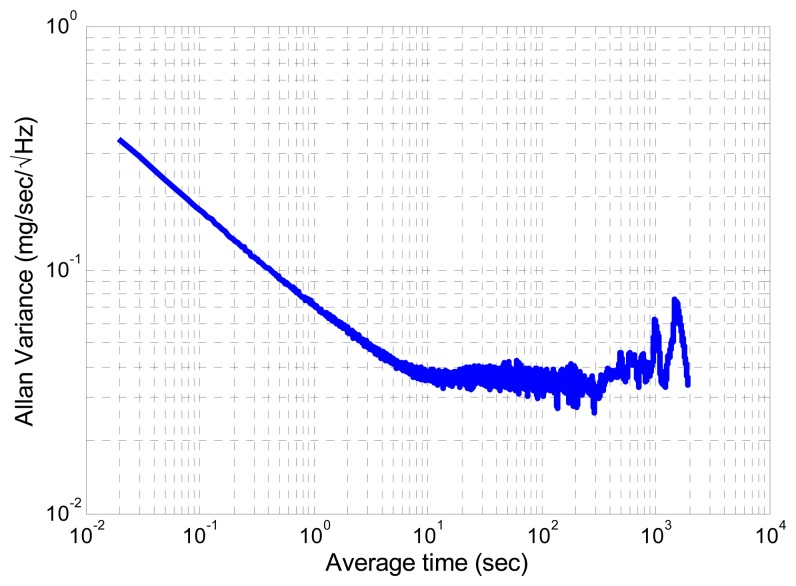
Allan variance of the second component of the accelerometer.

The sensor axis is non-orthogonally calibrated using a dedicated platform for aligning each axis with the gravity field and applying a least squares based estimation. An Allan variance study [20] is applied to the accelerometer signal, which was acquired during a static phase, for inferring the noise characteristics. Figure 3 shows the Allan variance results plotted for one of the sensor axes.

Three main noise components are identified: an angular random walk, given by the −½ slope part, the bias instability, given by the 0 slope curve part and the beginning of a ½ slope curve at high average time. Consequently, the accelerometer signal is modelled by:

(12) $$y_{a}=f_{ib}^{b}+b_{a}+n_{a}$$

where the three dimensional sensor signal **y***_a_* is composed by:

${\text{f}}_{ib}^{b}$ the specific force of the body frame with respect to the inertial frame, expressed in the body frame;
**b***_a_* is the bias of the accelerometer and;
**n***_a_* is a zero-mean Gaussian white noise with a standard deviation noted **σ***_a_*.

The bias **b***_a_* is assumed to follow a Gauss-Markov model whose parameters are determined with the Allan variance study. The bias is mathematically expressed by:

(13) $${\dot{b}}_{a}=\beta b_{a}+n_{b_{a}}$$

where **ḃ***_a_* is the acceleration bias derivative, β is a constant and **n_b_***__a__* is zero-mean Gaussian white noise with a standard deviation noted **σ***_b_a__*. The values of the accelerometer noise components are given in Table 1.

#### 3.2.3. Gyroscope

Similarly to the accelerometer signal, the gyroscope signal is studied over a long static phase and its Allan variance or spectral density is extracted. The angular rate is modeled by the following expression:

(14) $$y_{g}=\omega_{ib}^{b}+b_{\omega }+n_{\omega }$$

where:

**y***_g_*, the three-dimension signal of the gyroscope, is composed with $\omega_{ib}^{b}$ the angular rate of the body with respect to the inertial frame;
**b***_ω_* is the gyroscope bias equivalent to an angular drift and;
**n***_ω_* is a zero-mean Gaussian white noise with a standard deviation noted **σ***_ω_*.

**Figure 4.**
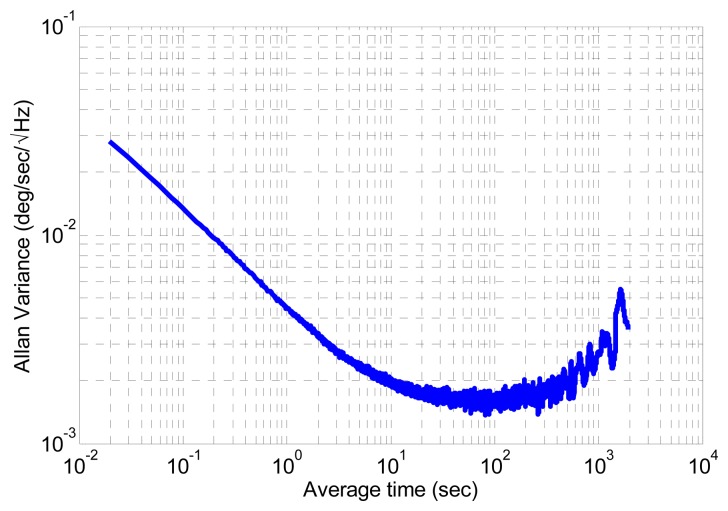
Allan variance of gyroscope signal third component.

The gyroscope bias is modelled as a random walk:

(15) $${\dot{b}}_{\omega }=n_{b_{\omega }}$$

where **ḃ***_ω_* is the gyroscope bias derivative and **n_b_***__ω__* is a zero-mean Gaussian white noise with standard deviation **σ***_b_ω__*. All deviations are extracted from the Allan variance plotted in Figure 4.

The angular rate $\omega_{ib}^{b}$ can be decomposed in two parts Equation (16) by introducing the navigation frame, which is the plane locally tangent to the Earth. The angular rate of the body frame with respect to the navigation frame $\omega_{nb}^{b}$ is given by:

(16) $$\omega_{ib}^{b}=\omega_{in}^{b}+\omega_{nb}^{b}$$

In the context of pedestrian navigation, the rotation of the navigation frame with respect to the inertial frame $\omega_{in}^{b}$ is rather small, as compared to the body dynamic expressed with $\omega_{ib}^{b}$. So $\omega_{in}^{b}$ is assumed to be a residual part embedded in the gyroscope bias **b***_ω_*. The noise component values are given in Table 2.

### 3.3. Gyroscope Quaternion

#### 3.3.1. Design of the Gyroscope Quaternion Model

Instead of using directly the gyroscope model, the gyroscope signal is interpreted as a rotation between two successive epochs. A mathematical model of this rotation, named the quaternion gyroscope, is proposed and demonstrated. This new model results from the integration of Equation (10) using the navigation and body frames:

(17) $$q_{b}^{n}(t+T_{s})=q_{b}^{n}(t)⊗q_{\omega }\left(t\right)$$

where:

**q_ω_** is the rotational quaternion between the two epochs *t* and *t* +*T_s_* defined in (18);
*T_s_* is the sampling period and;
$\omega_{nb}^{b}$ is the angular rate of the body frame with respect to the navigation frame. The latter is assumed to be constant over the period *t* to *t* +*T_s_*.

(18) $$q_{\omega }=f(\omega_{nb}^{b})=\left(\begin{array}{c} \cos\left(\frac{\left\|\omega_{nb}^{b}\right\|}{2}T_{s}\right)\\ \sin\left(\frac{\left\|\omega_{nb}^{b}\right\|}{2}T_{s}\right)\frac{\omega_{nb}^{b}}{\left\|\omega_{nb}^{b}\right\|} \end{array}\right)$$

The gyroscope quaternion is constructed similarly to Equation (18), but instead of being composed of $\omega_{nb}^{b}$, the quaternion gyroscope (noted **q_y_***__g__*) is composed of the gyroscope measurement (noted **y***_g_*):

(19) $$q_{{\text{y}}_{g}}=f(y_{g})$$

**Figure 5.**
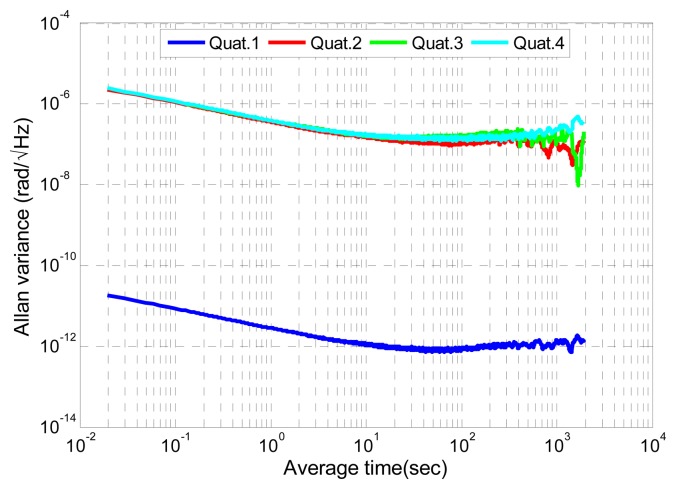
Allan variance of the fourth dimension quaternion gyroscope.

The quaternion **q_y_***__g__* is a rotational quaternion representing an approximation of **q_ω_**, which is the rotation between two successive epochs. Following previous sensor error modelling, an Allan variance study of **q_y_***__g__*, which has been created using all angular rates recorded during the static period, is conducted in order to assess the noise components of **q_y_***__g__*. The same trend is observed for all four components. The low average time part corresponds to white noise. Figure 5 shows also a bias instability and a correlated or a rate random walk.

Instead of modeling the gyroscope errors in the signal domain, it is performed in the quaternion set:

(20) $$q_{y_{g}}=q_{\omega }+b_{q_{\omega }}+n_{q_{\omega }}$$

where **n_q_***__ω__* is a zero-mean Gaussian white noise with standard deviation σ**_q_***__ω__*. The stochastic process chosen to model the gyroscope quaternion bias **b_q_***__ω__* is a random walk:

(21) $${\dot{b}}_{q_{\omega }}=n_{b_{q_{\omega }}}$$

where **ḃ_q_***__ω__* is the gyroscope quaternion bias derivative and **n_b_q__***___ω___* is a zero-mean Gaussian white noise with a standard deviation noted **σ_b_q__***___ω___*.

In order to a better understand the meaning of **b_q_***__ω__*, let us analyze the gyroscope quaternion **q_y_***__g__*. This quaternion corresponds to a rotation that approximates the true rotation **q_ω_** between two successive epochs. As a consequence, **b_q_***__ω__* appears to be the difference between both rotations. When it is small, it links the rotation **q_ω_** to **q_y_***__g__* by:

(22) $$q_{y_{g}}=q_{\omega }\left(I+{\bar{q}}_{\omega }⊗b_{q_{\omega }}\right)$$

where (*I* + **q̅_ω_**⊗**b_q_ω__**) represents an infinitesimal rotation linking the **q_ω_** to **q_y_***__g__*.

#### 3.3.2. Analysis of the Gyroscope Quaternion Bias

MAGYQ attitude angles estimation filter is based on an Extended Kalman Filter (EKF) whose working hypothesis (*H*) is that only white noise components are not modeled in the state vector. Because the proposed angular rate modeling in the quaternion set is new, previous EKF working hypothesis is now demonstrated for the Model (20). Two approaches are followed for the demonstration. The first analysis studies the physical meaning of the quaternion bias term when the latter is small. The second approach exploits simulated angular rates transformed into the quaternion set for analyzing the distribution of the noise terms.

##### Mathematical Derivation of the Gyroscope Quaternion

Assuming that the angular rates and the gyroscope measurements are small (<10 rad/s), in terms of amplitude over the sampling period *T_s_*, it is possible to re-write the expressions of the rotational quaternion **q***_ω_* and the gyroscope quaternion **q_y_***__g__*:

(23) $$\begin{array}{l} q_{y_{g}}=I_{q}+{\left(\frac{T_{s}}{2}y_{g}\right)}_{q}+\underset{T_{s}\to 0}{ο\left(T_{s}\right)}\\ q_{\omega }=I_{q}+{\left(\frac{T_{s}}{2}\omega_{nb}^{b}\right)}_{q}+\underset{T_{s}\to 0}{ο\left(T_{s}\right)} \end{array}$$

where **I***_q_* is the identity quaternion. $\underset{T_{s}\to 0}{o\left(T_{s}\right)}$ is a function that can be neglected when *T_s_* tends to zero.

Grouping both expressions in the gyroscope model Equation (14), the gyroscope quaternion **q_y_***__g__* becomes:

(24) $$q_{y_{g}}=q_{\omega }+{\left(\frac{T_{s}}{2}b_{\omega }\right)}_{q}+{\left(\frac{T_{s}}{2}n_{\omega }\right)}_{q}+\underset{T_{s}\to 0}{ο\left(T_{s}\right)}$$

with a first order approximation of Equation (24), only a random walk and a zero-mean white Gaussian noise terms remain in the last three components of **q_y_***__g__*. Indeed the noise of the first quaternion component equals zero and is removed in the MAGYQ filter. This observation is confirmed by the small value that is observed in the Allan Variance plot (Figure 5). This mathematical derivation ends the first demonstration and proves that hypothesis *H* is verified at the first order.

##### Validation of the Gyroscope Quaternion Modeling by Simulation

Angular rates are simulated in order to demonstrate the validity of the gyroscope quaternion model proposed in Equations (20) and (21). The signal **ỹ***_g_* is simulated for a static phase. Consequently it is only composed of a zero-mean white noise and a random walk. Using the simulated gyroscope signal and Equation (19), it is possible to construct the corresponding gyroscope quaternion **q_ỹ_***__g__*. A first comparison of the simulated angular rates and the gyroscope quaternions is performed in the frequency domain for assessing the similarities.

**Figure 6.**
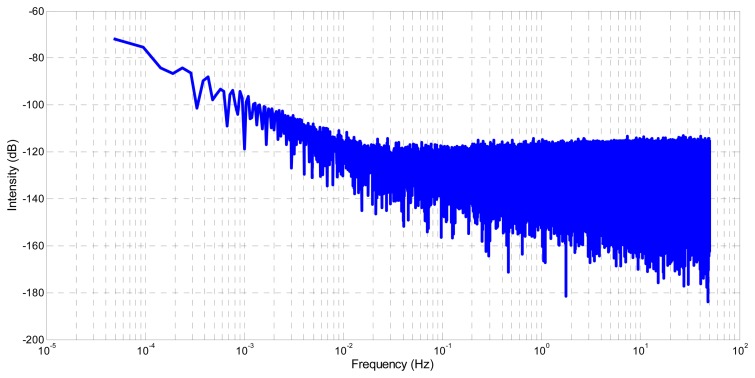
Spectral density of the simulated gyroscope signal **ỹ***_g_*.

**Figure 7.**
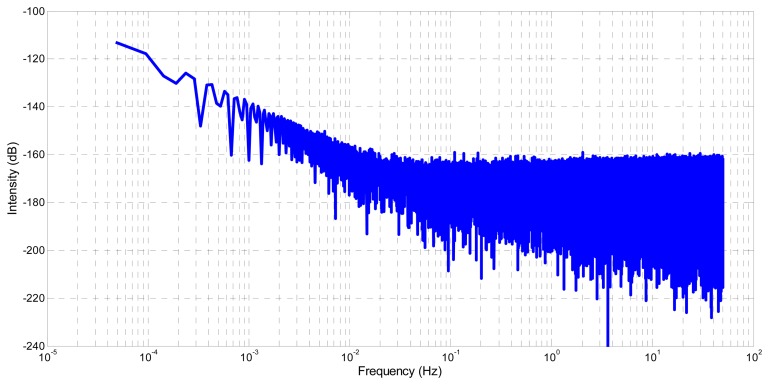
Spectral density of gyroscope quaternion **q_ỹ_***__g__*.

The spectral density, computed with Fast Fourier Transform [21,22], is used to analyze the noise terms embedded in **q_ỹ_***__g__*. Figures 6 and 7 show respectively the spectral density of **ỹ***_g_* and **q_ỹ_***__g__*. The same trend is observed in both cases. At low frequency, a curve with a slope of −20 db/decade, which is characteristic of a random walk, is visible. At high frequency, a flat curve slope characterizing a white noise is also visible. These similarities validate the correctness of the proposed gyroscope quaternion modelling.

In order to test if hypothesis *H* is verified with the proposed model, a Kolmogorov-Smirnov test [23] is conducted to evaluate the distance between two cumulative distribution functions (CDF). The first CDF is computed with **q_ỹ_***__g__* over 100,000 samples. The second CDF is computed with the simulated gyroscope quaternions **q̃_y_***__g__* using the Model (20) over the same time interval. The white noise and random walk stochastic processes parameters are extracted from the Allan Variance analysis previously detailed.

The hypothesis *H*_0_ under test is that “the two signals are following the same distribution law”. In order to be unaffected by the choice of stochastic parameters, the distributions are centered and normalized. The Kolmogorov-Smirnov test is performed considering a 5% risk of wrongly rejecting the hypothesis:

(25) $$T=\text{proba}\left(\underset{k}{\sup}\left(\left|D_{1}\left(k\right)-D_{2}\left(k\right)\right|\right)\geq \frac{1.36}{\sqrt{N_{\text{samples}}}}\right)$$

*H*_0_ is verified with a probability greater than 80% for the second, third and fourth quaternion elements. Only the first component of the quaternion slightly fails the hypothesis *H*_0_. However the very low noise level of this term justifies the chosen model, *i.e.*, white noise and random walk.

From this analysis, it can be concluded that the proposed quaternion based error model is correctly capturing non-stationary noises and only white noise remains. Consequently, designing an EKF for estimating the orientation of the body frame with respect to navigation frame using the proposed novel quaternion based angular rates model is justified.

## 4. Dynamic State Propagation

MAGYQ, the attitude and heading estimation filter, is now described. The state vector is first presented and followed by the initialization process. Finally, the mathematical expression of the state propagation is described.

### 4.1. State Vector

The complete state vector includes the quaternion form $q_{b}^{n}$ of the rotation between the body and the navigation frame, but also all sensor error sources: the gyroscope quaternion bias **b_q_***__ω__* and the accelerometer bias **b***_a_*. The state vector **x** is:

(26) $$x^{T}=\left[\begin{array}{ccc} q_{b}^{nT} & {b_{q_{\omega }}}^{T} & {b_{a}}^{T} \end{array}\right]$$

### 4.2. Initialization

The initial rotation is computed using the accelerometer and magnetometer measurements during a static phase without magnetic disturbances. During the initialization, it is expected that the sensors are directly measuring the gravity and the Earth magnetic fields.

### 4.3. Error State Propagation Model

The additive error state vector δ**x** links the estimated state vector noted **x̂** to the true state vector noted **x**, defined in Equation (26), by:

(27) $$x=\hat{x}+\delta x$$

The evolution laws of the acceleration and gyroscope quaternion biases are those of the random walk stochastic process. The discrete equations with a first order approximation are:

(28) $$\left\{ \begin{array}{l} b_{q_{\omega }}(t+T_{s})=b_{q_{\omega }}(t)+T_{s}n_{b_{q_{\omega }}}(t)\\ b_{a}(t+T_{s})=\left(1-\beta T_{s}\right)b_{a}(t)+T_{s}n_{b_{a}}(t) \end{array}\right.$$

The propagation of the rotation ${\text{q}}_{b}^{n}$ is given by the integration of Equation (10):

(29) $$q_{b}^{n}(t+T_{s})=q_{b}^{n}(t)⊗q_{\omega }\left(t\right)$$

The quaternion **q_ω_** is unknown, only an approximation noted **q̂_ω_** is accessible through the gyroscope quaternion and the estimated bias:

(30) $${\hat{q}}_{\omega }=q_{y_{g}}-{\hat{b}}_{q_{\omega }}$$

The estimated rotation ${\hat{q}}_{b}^{n}$ is propagated using the estimated rotation between two successive epochs **q̂_ω_**:

(31) $${\hat{q}}_{b}^{n}(t+T_{s})={\hat{q}}_{b}^{n}(t)⊗{\hat{q}}_{\omega }\left(t\right)$$

In this step, ${\hat{q}}_{b}^{n}$ and **q̂_ω_** are assumed to be rotational quaternions, so they must satisfy $\left\|{\hat{q}}_{b}^{n}\right\|=\left\|{\hat{q}}_{\omega }\right\|=1$ A normalization step is applied. In the remaining equations, all quaternion components ( )**_q_** are normalized to comply with the rotation subset of the quaternion set. With a first order development of Equations (28) and (29), the state error model becomes:

(32) $$\delta x\left(t+T_{s}\right)=\left(\begin{array}{ccc} C_{{\hat{q}}_{\omega }} & -M_{{\hat{q}}_{b}^{n}} & 0\\ 0 & I & 0\\ 0 & 0 & \left(I-\beta T_{s}\right) \end{array}\right)\delta x(t)+\left(\begin{array}{ccc} -M_{{\hat{q}}_{b}^{n}} & 0 & 0\\ 0 & T_{s}I & 0\\ 0 & 0 & T_{s}I \end{array}\right)\left(\begin{array}{c} n_{q_{\omega }}(t)\\ n_{b_{q_{\omega }}}(t)\\ n_{a}(t) \end{array}\right)$$

Given that the first component of the state vector is the orientation of the body frame with respect to the navigation frame and that the second component is the gyroscope quaternion bias, Equation (32) shows the correlation between these two components. It indicates that the error in the orientation comes from the precedent error combined with the rotation error between two successive epochs.

## 5. Static Period Detection Threshold

All updates are based on *a priori* knowledge of opportune local fields in the navigation frame. As it will be explained in next part, static acceleration or magnetic field in the navigation frame informs about the attitude angles and can be used to correct the state parameters. But the challenge is that the data are only available in the body frame. A reference field must be found for inferring the presence of a static field period in the navigation frame using the measurements in the body frame.

By definition, the vector norm is invariant through isometric transformation. Consequently, the static period detection process is primarily based on the study of the norm. It consists in a statistical test of the field time derivative, such as likelihood ratio test (LTR) [24]. It performs a comparison with a predetermined threshold [16] and rejects the static hypothesis if the innovation is too large. Last approach is known as adaptive Kalman Filtering with Fault Detection and Exclusion (FDE) technique. In all cases, the goal is to identify if at current epoch, the acceleration or magnetic field in the navigation frame is constant.

At epoch *t*, the static field detector is based on the study of the variance of the field of interest and a comparison with an initially fixed threshold:

(33) $$\frac{1}{N+1}\sum_{k=t-NT_{s}}^{t+T_{s}}{\left(\left\|{\mathrm{field}}^{b}(k)\right\|-\xi_{\text{ref}}\right)}^{2}<\gamma_{1}$$

where **field** refers to the acceleration or magnetic field. *N* is the length of the static period. ξ*_ref_* is the reference norm for the current static period and γ_1_ is a threshold.

The reference norm is defined at the beginning of the static period as the mean of the *N_first_* first values of the static period. The threshold γ_1_ is defined as the deviation of the field during a static and undisturbed period. This first detector compares a global deviation with a threshold. In this approach, each sample data must be close to the reference norm, which is a limiting factor. Consequently a variant is introduced:

(34) $$\left|\left\|{\mathrm{field}}^{b}(k)\right\|-\xi_{\text{ref}}\right|\leq \gamma_{2}$$

where γ_2_ is a threshold different from γ_1_ but also fixed during a static and undisturbed period. This threshold is used to reject outliers. It completes the first detector dedicated to the global study of deviation over the current static period.

## 6. Magnetometer and Accelerometer-Based Heading and Bias Correction

The use of accelerometers and magnetometers can assist the heading estimation problem. In the literature, it is well known that if known static fields are sensed, e.g., Earth magnetic field or Earth gravity field, a direct observation of the angles is possible. The situation is more challenging if the measured fields are unknown. Even in this case, it is still possible to extract heading information by correcting the angular drift modelled in the gyroscope quaternion bias. This is now explained.

### 6.1. Quasi-Static Field (QSF) Error Model

Two similar Quasi-Static Field (QSF) error models are proposed for the magnetic field and the acceleration field. They translate novel observations to correct the state vector when conditions (33) and (34) are verified, *i.e.*, the acceleration or magnetic field is assumed to be constant in the navigation frame. The two possible QSF updates are now explained, starting with the quasi-static magnetic field error model and continuing with the quasi-static acceleration field.

Let us consider the *k*-th quasi-static period of the magnetic field with a reference field noted ${\text{m}}_{ref_{k}}^{n}$. At each epoch of the present static period, the sensed magnetic field **y***_m_* is transformed into the navigation frame using the estimated rotation ${\hat{q}}_{b}^{n}$ and compared to the reference field. The innovation *δ***z***_m_* obtained by this correction is:

(35) $${\left(\delta z_{m}\right)}_{q}={\left(m_{ref_{k}}^{n}\right)}_{q}-{\hat{q}}_{b}^{n}⊗{\left(y_{m}^{b}\right)}_{q}⊗{\bar{\hat{q}}}_{b}^{n}$$

For the acceleration field, the principle is the same, but the accelerometer measures **y***_a_* and gives a biased estimation of the acceleration field, so the acceleration bias must be removed. Let ${\text{f}}_{ref_{k}}^{n}$ be the reference specific force at the *k*-th quasi-static period. This period is not necessarily the same as the quasi-static magnetic field period. The innovation noted δ**z***_a_* is:

(36) $${\left(\delta z_{a}\right)}_{q}={\left(f_{ref_{k}}^{n}\right)}_{q}-{\hat{q}}_{b}^{n}⊗{\left(y_{a}^{b}-{\hat{b}}_{a}\right)}_{q}⊗{\bar{\hat{q}}}_{b}^{n}$$

One of the difficulties is to determine the reference field. In the first case, the norm of the field equals the norm of the Earth gravity field or the Earth magnetic field. In this case, there is a global correction of the orientation. But in indoor environments, perturbed by magnetic disturbances, only few such cases occur. The second case uses any quasi-static field as long as the Earth magnetic field disturbances are evaluated as constant. The reference field is then computed as the mean of the *N_first_* values of the field of interest. The QSF error models are derived with a first order development of Equations (35) and (36):

(37) $$\left\{ \begin{array}{l} \delta z_{m}=\left[\begin{array}{ccc} h_{1}\left({\hat{q}}_{b}^{n},m_{ref_{k}}^{b}\right) & 0 & 0 \end{array}\right]\delta x-h_{2}\left({\hat{q}}_{b}^{n}\right)n_{m}\\ \delta z_{a}=\left[\begin{array}{ccc} h_{1}\left({\hat{q}}_{b}^{n},a_{ref_{k}}^{b}\right) & 0 & -h_{2}\left({\hat{q}}_{b}^{n}\right) \end{array}\right]\delta x-h_{2}\left({\hat{q}}_{b}^{n}\right)n_{a} \end{array}\right.$$

The functions *h*_1_ and *h*_2_ are defined by:

(38) $$\begin{array}{l} h_{1}:\left(q,x\right)\in \left(ℍ,{\mathbb{R}}^{3}\right)\mapsto 2\left[\begin{array}{cc} q_{1}x+\left[u_{q}\times \right]x & \langle u_{q},x\rangle I_{3\times 3}-\left[\left(q_{1}x+\left[u_{q}\times \right]x\right)\times \right] \end{array}\right]\\ h_{2}:q\in ℍ\mapsto \left({q_{1}}^{2}-{\left\|u_{q}\right\|}^{2}\right)I_{3\times 3}+2u_{q}\cdot {u_{q}}^{T}+2q_{1}\left[u_{q}\times \right] \end{array}$$

### 6.2. Magnetic Angular Rate and Acceleration Gradient Updates

Magnetic angular rate update (MARU) or acceleration gradient update (AGU) are applied during QSF periods. When the field is constant in the navigation frame, its variation between two successive epochs in the body frame is only due to the body frame rotation with respect to the navigation frame:

(39) $${\left({\mathrm{field}}^{b}(t)\right)}_{q}=q_{\omega }\left(t\right)⊗{\left({\mathrm{field}}^{b}\left(t+T_{s}\right)\right)}_{q}⊗{\bar{q}}_{\omega }\left(t\right)$$

where **q_ω_** is defined in Equation (18).

Similarly to the precedent part on QSF, two applications of this update are possible. The first is the magnetic angular rate update and the second is the acceleration gradient update.

#### 6.2.1. Magnetic Angular Rate Model

The evolution law of the magnetic field, expressed in body frame, during a quasi-static magnetic field period *k*, is derived from Equation (39). The magnetic field **m̂** at epoch *t* + *T_s_* is estimated from the magnetic field sensed at epoch *t* and the estimated rotation **q̂***_ω_* between the epochs *t* and *t* + *T_s_*:

(40) $${\left({\hat{m}}^{b}(t+T_{s})\right)}_{q}={\bar{\hat{q}}}_{\omega }\left(t\right)⊗{\left(y_{m}^{b}\left(t\right)\right)}_{q}⊗{\hat{q}}_{\omega }\left(t\right)$$

This estimated value is compared to the magnetometer data at epoch *t* + *T_s_*, which gives the magnetic angular rate innovation δ**z***_MARU_*:

(41) $$\delta z_{\text{MARU}}=y_{m}^{b}\left(t+T_{s}\right)-{\hat{m}}^{b}(t+T_{s})$$

A first order development of Equation (41) gives:

(42) $$\delta z_{\text{MARU}}=\left[\begin{array}{ccc} 0 & -h_{3}\left({\hat{q}}_{\omega },m^{b}(t)\right) & 0 \end{array}\right]\delta x+\left[\begin{array}{cc} I_{3\times 3} & -h_{2}\left({\bar{\hat{q}}}_{\omega }\right) \end{array}\right]\;\left[\begin{array}{c} n_{m}\left(t+T_{s}\right)\\ n_{m}\left(t\right) \end{array}\right]$$

where the function *h*_2_ is defined in Equation (38) and the function *h*_3_ is given by

(43) $$h_{3}:\left(q,x\right)\in \left(ℍ,{\mathbb{R}}^{3}\right)\mapsto 2\left[\begin{array}{cc} q_{1}x-\left[u_{q}\times \right]x & \langle u_{q},x\rangle I_{3\times 3}+\left[\left(q_{1}x-\left[u_{q}\times \right]x\right)\times \right] \end{array}\right]$$

This update enables to correct the angular drift modelled in the gyroscope quaternion bias.

#### 6.2.2. Acceleration Gradient Model

Similarly to the magnetic angular rate update, the acceleration gradient update is based on identified QSF. During a static phase of the acceleration field, its evolution at epoch *t* + *T_s_* is predictable using the rotation between two epochs and the acceleration field at epoch *t*. Following this approach, the acceleration field is estimated by:

(44) $${\left({\hat{f}}^{b}(t+T_{s})\right)}_{q}={\bar{\hat{q}}}_{\omega }\left(t\right)⊗{\left(y_{a}^{b}\left(t\right)-{\hat{b}}_{a}\left(t\right)\right)}_{q}⊗{\hat{q}}_{\omega }\left(t\right)$$

The acceleration gradient innovation δ**z***_AGU_* is finally computed by

(45) $$\delta z_{\text{AGU}}=y_{a}^{b}\left(t+T_{s}\right)-\left({\hat{f}}^{b}(t+T_{s})+\left(I-\beta T_{s}\right){\hat{b}}_{a}\left(t\right)\right)$$

In Equation (45), the acceleration bias term **b̂***_a_* (*t*) is added to the specific force **f̂***^b^* for providing a comparison with the acceleration ${\text{y}}_{a}^{b}$. With a first order development, the acceleration gradient model becomes:

(46) $$\delta z_{\text{AGU}}=\left[\begin{array}{ccc} 0 & -h_{3}\left({\hat{q}}_{\omega },y_{a}^{b}\left(t\right)-{\hat{b}}_{a}\left(t\right)\right) & I-\left(\beta T_{s}+h_{2}\left({\bar{\hat{q}}}_{\omega }\right)\right) \end{array}\right]\delta x+\left[\begin{array}{cc} I_{3\times 3} & -h_{2}\left({\bar{\hat{q}}}_{\omega }\right) \end{array}\right]\;\left[\begin{array}{c} n_{a}\left(t+T_{s}\right)\\ n_{a}\left(t\right) \end{array}\right]$$

where the functions *h*_2_ and *h*_3_ are defined in Equations (38) and (43). This update offers very interesting observations since it involves both the accelerometer and gyroscope quaternion biases.

## 7. Step Length Estimation

Following a PDR approach, step lengths and headings are merged for recursively propagating the pedestrian's position at epoch *t* + Δ*T* from the one at epoch *t*. Whereas the headings are computed using MAGYQ, the step lengths are computed following a three steps process [25]. First, the motion mode, the handheld device carrying mode and the step frequency *f_step_* are estimated based on a decision tree and several features extracted from time and frequency domain analyses of the accelerations and angular rates. Step events are estimated in a separated independent process that is adaptive to the pedestrian walking pace. Finally, the step lengths are estimated with the following model:

(47) $$s=h\left(af_{\text{step}}+b\right)+c$$

The step length *s* is a function of the step frequency *f_step_*, the user's height *h* and three parameters {*a*,*b*,*c*}. These parameters are estimated using a least square method based on known users' walking speed that is determined with an accurate differential GPS positioning technique. This satellites signal based solution is also the reference for the experimental tests that are now presented.

## 8. Experimental Validation

Experimental tests are conducted for evaluating the performances of the MAGYQ attitude estimation filter. After a description of the experimental protocol, the filter outcomes are assessed in terms of orientation and position.

### 8.1. Description of the Experiment

The IMU used for the experiment is the ADIS 16488 [26]. This inertial and magnetic unit is composed by a tri-axis magnetometer, a tri-axis accelerometer and a tri-axis gyroscope. All sensors are co-aligned and their measurements are expressed in a unique common frame: the body frame. The IMU is connected to an acquisition device that registers the sensor data at 100 Hz for an offline processing.

Three people, whose age is between 25 and 56 years and height varied between 1.62 and 1.84 m, were equipped with this hardware. During the data acquisition, they kept the body frame roughly fixed with respect to it. This means that the sensor pointing direction is approximately the same as the walking direction and no misalignment is considered in this paper. The three test subjects are labelled M1, M2 and W1 (where M is for man and W for woman), in the following figures. The total walking path length was about 1.5 km including successive periods in outdoor and indoor environments. The experiment starts outdoors with the magnetometer calibration and the initialization phase. This initialization must be performed in surroundings without magnetic disturbances where only the earth magnetic field is measured. The people continued the tests walking successively in the parking lot and inside a building.

**Figure 8.**
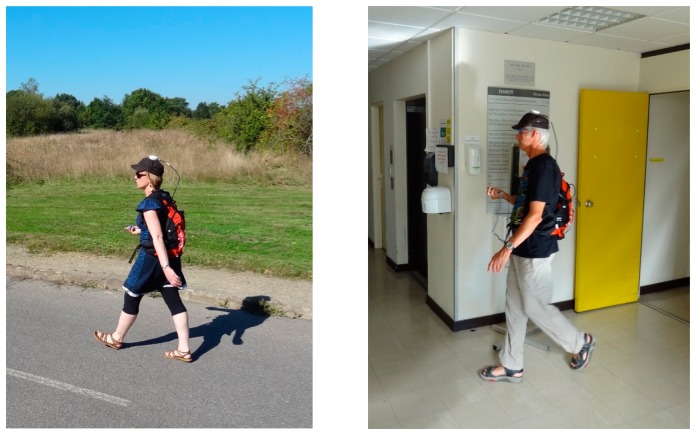
Experimental test equipment.

To evaluate the performances of the present algorithm, a reference system is needed. In outdoor spaces, where Global Navigation Satellite Signals (GNSS) are available, a post-processed differential trajectory was calculated using GrafNav software from NovAtel [27]. The 5 cm accurate reference solution is post-processed at 5 Hz for the GNSS antenna attached on the pedestrian's cap, as shown in Figure 8. The GNSS reference trajectory is only available in outdoor spaces. Inside the building, the main corridor directions are used. Let us notice that the width of these corridors is small and the subjects walked along these directions. The latter are extracted from an accurate office map. The performances are analyzed with the different footpaths overlayed on the building background map. It is important to recall that even outside, the results are only computed using the handheld MEMS signals and the proposed MAGYQ filter. The same MEMS signals are used for all tests.

### 8.2. Performance Assessment of Estimated Angles

Prior to assessing MAGYQ performances combined with step lengths in the positioning domain, the attitude angles estimation is evaluated with two different tests:

- Indoor data collection with static IMU.
- Dynamic outdoor data collection.

#### 8.2.1. Indoor Static Test

**Figure 9.**
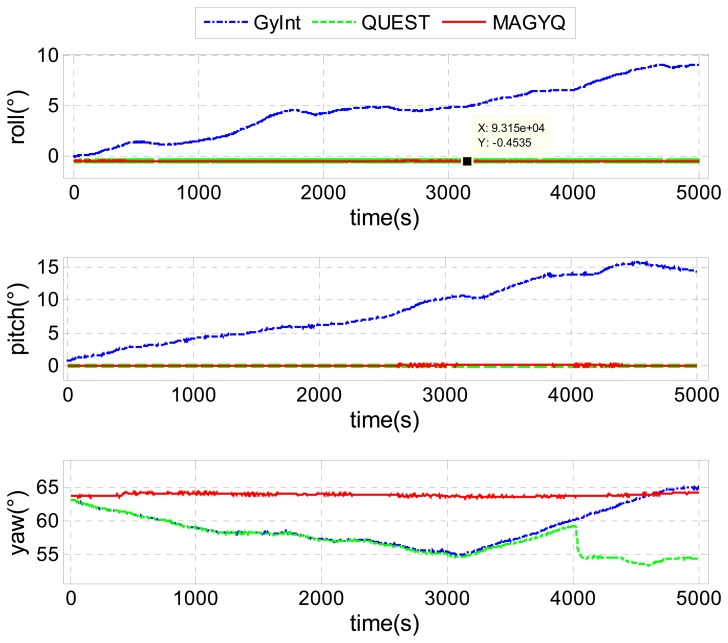
Static test.

For the static test, 5000 seconds of static IMU data were collected in an indoor space, where the local magnetic field is constant but different from the earth magnetic field. Three attitude estimators are applied to the recorded data. Two of them are state of the art estimators. All angle outcomes are shown in Figure 9.

The angles in blue are estimated with the **GyInt** approach, for Gyroscope Integration. This state of the art estimator corresponds to a nominal gyroscope bias corrected integration only. In this strategy, gyroscope measurements are corrected with the nominal angular rate bias, extracted from the Allan Variance analysis, and then propagated in time. It can be observed that GyInt attitude estimator presents a large angular random walk drift.

The second estimator, shown in green, corresponds to the well-known **QUEST** estimator. Corrections are issued from the detection of the earth gravity or earth magnetic field. In this indoor configuration, only the earth gravity measurement hypothesis is verified. Consequently, only roll and pitch angles are corrected. The yaw angle is not observable and becomes badly corrected. A norm, close to the geomagnetic field, is detected and biases the solution.

The last estimator, shown in red, is the **MAGYQ** filter. Contrary to the previous two estimators, it can be observed that the attitude is continuously corrected even if the sensed magnetic field is not the earth magnetic field. As expected, MAGYQ is able to absorb the angular drift into the quaternion gyroscope bias even with “perturbed magnetic field” as long as they are constant. The attitude is found to be stable over the 1 h and 23 min period.

#### 8.2.2. Dynamic Outdoor Data Collection

**Figure 10.**
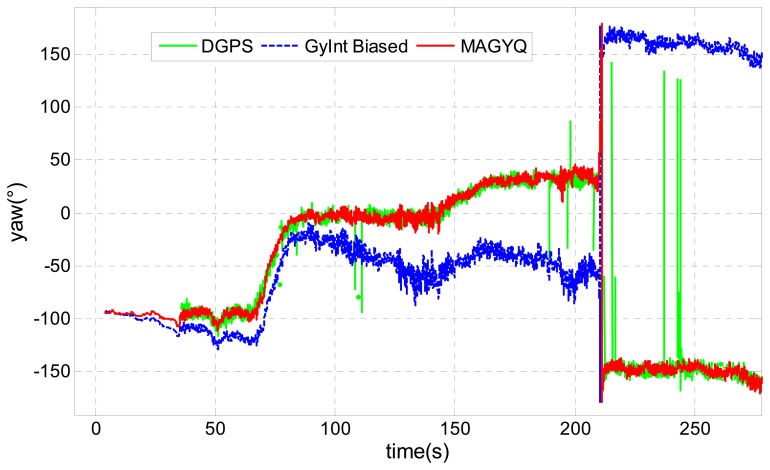
Static test.

The test consists in an outdoor walk without changing misalignment between the walking direction and the sensor pointing direction. The aim of this test is to verify if MAGYQ filter corrects the initial gyroscope bias, even if the latter is wrongly calibrated. The reference heading, shown in green in Figure 10, is post-processed using the Differential GPS (DGPS) solution.

It is important to mention that some outliers are present in the GPS positions, leading to some inaccurate heading estimates. These outliers are principally due to satellite signals multi-path and fading effects induced by the surrounding vegetation and buildings. Globally, the DGPS solution provides an accurate estimation of the heading at a lower rate than the IMU one. GyInt estimator is also applied but the initial bias used to correct the angular rates is slightly exaggerated in order to better observe the performance of MAGYQ filter. This is the reason why it is labelled GyInt Biased and depicted in blue in the same figure.

An important time drift of the yaw angle is observed with this approach. On the contrary, MAGYQ filter, depicted in red, provides an important correction of the gyroscope quaternion bias, which deletes the angular drift. Because MAGYQ filter takes advantages of accelerometer and magnetometer signals for correcting the gyroscope and accelerometer drifts in much more situations than the other filters, the observed angle estimation performances are better. The heading is continuously readjusted and its estimation error remains below 10° over the 1.5 km walk. This is computed using the DGPS heading during the 15 min period.

### 8.3. Performance Assessment of Estimated Pedestrian Trajectory

**Figure 11.**
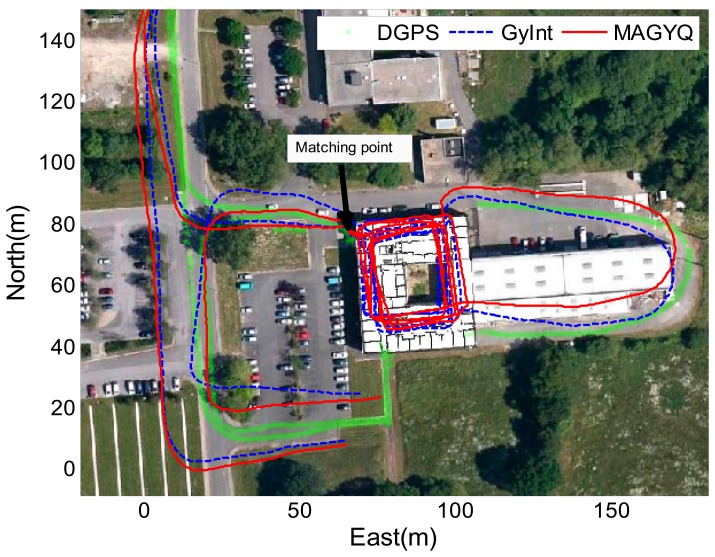
Walking paths of M1.

**Figure 12.**
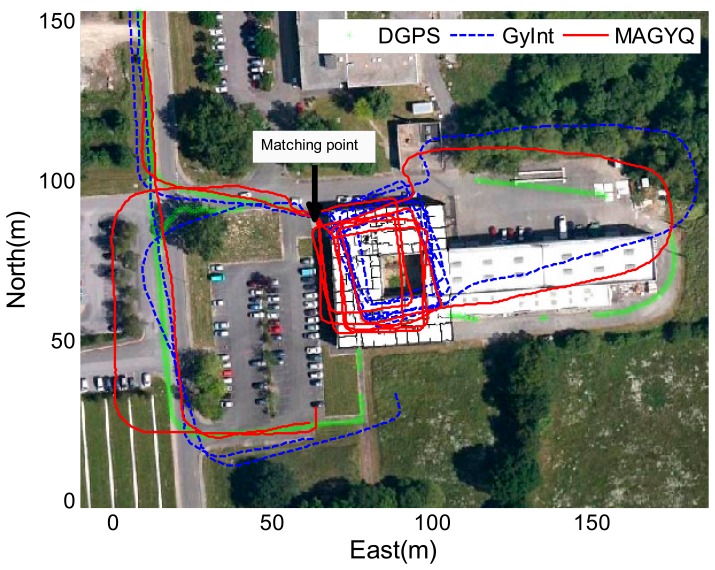
Walking paths of W1.

**Figure 13.**
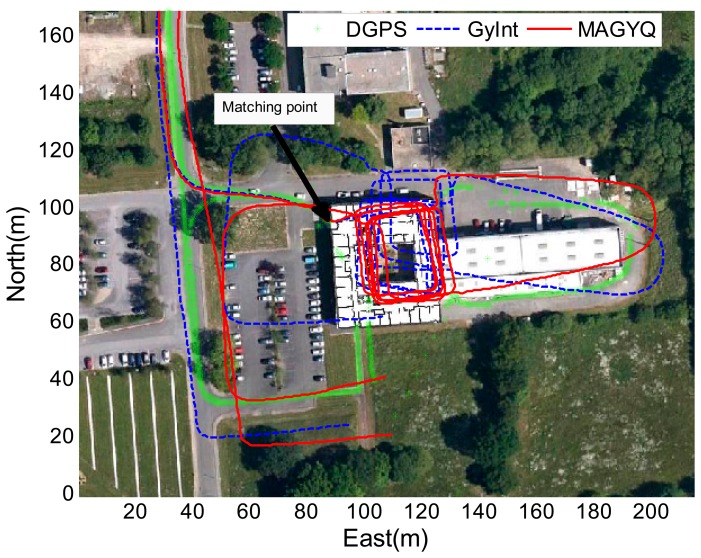
Walking paths of M2.

After verifying the filter behavior, the MAGYQ based heading is mixed with step length for estimating the PDR footpath. Figures 11, 12 and 13 show the three trajectories corresponding to the test described in Section 8.1. The outdoor reference is given by the DGPS solution and is plotted in green.

The GyInt and MAGYQ inertial trajectories are estimated using the same step length calculated with the Model (47) and individually calibrated {*a*,*b*,*c*} parameters. The trajectory estimation only differs in the heading process. The GyInt estimation (blue) is performed by integrating the angular rates, knowing the nominal gyroscope bias. The red trajectory corresponds to the positions estimated using the heading from MAGYQ outcomes. A matching point is applied to initialize the free-inertial footpath using the DGPS position calculated at the entrance of the building. This enables to assess the heading estimation quality inside the buildings using the main corridor directions.

In Figure 11, the gyroscope propagation (GyInt) solution is quite accurate and there is no significant difference with MAGYQ estimation. In this case, the gyroscope quaternion bias remains relatively stable. But in Figures 12 and 13, GyInt solution is drifting over the entire walking path, whereas MAGYQ algorithm is able to correct a substantial part of the drift. It is observed that the step length errors are dominating the positioning error budget.

## 9. Conclusions/Outlook

A novel Magnetic, Acceleration fields and GYroscope Quaternion (MAGYQ) based attitude and heading estimation filter is proposed and demonstrated with handheld sensors. This filter parameterizes the state vector directly in the quaternion set in order to reduce the filter error propagation and some of the linearization issues following an Extended Kalman Filter approach. Instead of using a gyroscope signal modeling at the signal level, a new quaternion based model is proposed introducing the gyroscope quaternion bias term. The compatibility of this new angular rate modeling with the Extended Kalman Filter underlying assumptions is demonstrated at the theoretical level and with a simulation.

Among the novel aspects are the introduction of new observation equations that enable to correct the drifting part of the acceleration and gyroscope data using the gradient of the observed fields. A new quaternion set based magnetic angular rate (MARU) update is proposed. It is applied whenever the local magnetic field, perturbed or not, is identified as constant over a sliding window. This update is frequently applied along the pedestrian's route and is found to be very powerful for estimating the gyroscope drift even inside buildings. Whenever the acceleration is found to be steady over a time interval, a new quaternion based acceleration gradient update (AGU) is also proposed. Through this update, not only is the acceleration bias observed but also the gyroscope quaternion bias.

Outcomes of the experimental tests assess the good performance of MAGYQ in correctly estimating the attitude angles as compared to a differential GPS solution. The evaluation is also performed during static acquisition conditions. A heading error below 10° over a 1.5 km walk is observed for two man and one woman test subjects. The results are also compared with the well-known QUEST algorithm and the GyInt algorithm, *i.e.*, a gyroscope integration scheme with initial bias correction. MAGYQ is found to provide better results than the other approaches, especially in indoor space where the earth magnetic field is disturbed and the other strategy fail to mitigate the drifting noise components. The heading estimated with MAGYQ is also combined with step lengths estimated with an individually calibrated model for observing the performance in the positioning domain. Direct comparison with background building map is finally proposed. It is observed that step length errors are dominating the positioning error. No misalignment between the pedestrian walking direction and the handheld device pointing direction is considered in this research, this aspect belongs to future research perspectives.

## Figures and Tables

**Table 1.** Accelerometer noise parameters.

	**Velocity Random Walk**	**Bias Instability**	**Correlated Noise**	

	**Allan Deviation (mg/**√**Hz)**			**Correlated Time (s)**

Axis *X*	0.0650	0.1223	0.1484	18.16
Axis *Y*	0.0717	0.0326	0.0373	48.05
Axis *Z*	0.0639	0.0626	0.0881	197.2

**Table 2.** Gyroscope noise parameters.

	**Angular Random Walk**	**Bias Instability**	**Rate Random Walk**

	**Allan Deviation (rad/s/**√**Hz)**		

Axis *X*	0.00413	0.00117	0.00158
Axis *Y*	0.00445	0.00121	0.00142
Axis *Z*	0.00442	0.00105	0.00157
